# Evaluation of radiation therapy on grafted and non-grafted defects: an experimental rat model

**DOI:** 10.1590/1678-7757-2024-0211

**Published:** 2025-01-13

**Authors:** Milena Suemi IRIE, Isabella Neme Ribeiro dos REIS, Luiz Gustavo Gonzáles OSUNA, Guilherme José Pimentel Lopes de OLIVEIRA, Rubens SPIN-NETO, Priscilla Barbosa Ferreira SOARES

**Affiliations:** 1 Universidade Federal de Uberlândia Faculdade de Odontologia Departamento de Periodontia e Implantodontia Uberlândia Brasil Universidade Federal de Uberlândia, Faculdade de Odontologia, Departamento de Periodontia e Implantodontia, Uberlândia, Brasil.; 2 Universidade de São Paulo Faculdade de Odontologia Departamento de Estomatologia São Paulo Brasil Universidade de São Paulo, Faculdade de Odontologia, Departamento de Estomatologia, São Paulo, Brasil.; 3 Aarhus University Section for Oral Radiology Department of Dentistry and Oral Health Aarhus Denmark Aarhus University, Section for Oral Radiology, Department of Dentistry and Oral Health, Health, Aarhus, Denmark.

**Keywords:** Radiation ionizing, Bone, Bone substitutes, X-ray microtomography, Collagen

## Abstract

**Objective:**

This study aimed to assess the effects of a single-dose radiation therapy (15 Gy) on grafted and non-grafted defects, bone microarchitecture, and collagen maturity.

**Methodology:**

Bone defects were surgically created in rat femurs. The right femur defect was filled with blood clot (group “Clot”) and the left femur defect by deproteinized bovine bone mineral graft (group “Xenograft”). The animals were divided into two groups: without radiation therapy (nRTX) and with radiation therapy (RTX). Microtomographic (bone volume fraction, BV/TV; trabecular thickness, Tb.Th; trabecular number, Tb.N; trabecular separation, Tb.Sp), histological, and histomorphometric analyses were performed 14 days after the surgery. Two-way ANOVA with Tukey post hoc test was used to compare the groups (α=5%).

**Results:**

Microtomographic analysis revealed that radiation therapy led to smaller BV/TV and Tb.N in both Clot and Xenograft groups. Regardless of radiation therapy, defects filled with xenografts showed a larger Tb.N. In contrast, the Clot group demonstrated increased BV/TV and Tb.Th. The histomorphometric results were consistent with those obtained by microtomography. Intermediately and densely packed collagen were predominant among the groups. Histological analysis revealed disorganized bone formation bridging the cortical borders of the lesions in the RTX group. The involvement of primary bone with graft particles was commonly observed in all xenograft groups, and radiation therapy did not affect the percentage of bone-graft contact.

**Conclusion:**

Single-dose radiation therapy affected bone repair, resulting in a smaller amount of newly formed bone in both grafted and non-grafted defects.

## Introduction

Radiation therapy is widely used in radiation oncology, i.e., cancer treatment. Although tumor irradiation improves patient survival, it also adversely affects healthy tissues. Thus, ionizing radiation to the bone might affect its volume, composition, and microarchitecture.^1^ Additionally, both the organic and mineral components of the matrix are damaged.^1^ It has been demonstrated that even low-dose radiation may induce osteoclastogenesis due to stimulatory inflammatory cytokines in the surrounding irradiated tissue. Concomitant decrease in osteoblast activity and increase in osteoclast activity result in greater bone resorption and increased trabecular bone turnover.^2^ These effects are dose- and time-dependent.^3^ Bone cell apoptosis, mostly osteoblasts and osteocytes, results in reduced bone density, impairing the natural morphology and inner properties of bone. Osteoradionecrosis (ORN) is one of the most severe complications of head and neck radiation therapy. The narrowing of the blood vessels leads to hypocellular tissue, hypoxia, and bone necrosis. Tooth extraction after radiation therapy is a risk factor for ORN. Extraction and surgical intervention before radiation therapy have also been suggested.^4^

The treatment of squamous cell carcinoma usually requires surgical resection and radiation therapy, leading to functional maxillofacial sequelae. Prosthetic rehabilitation with dental implants may be an option for patients experiencing considerable tissue loss. In this context, the timing of dental implant placement remains controversial. Despite the risk of ORN, some studies have shown the viability of dental implant placement at a delayed stage (after radiation therapy).^5^ However, to avoid a second intervention, implant placement has more recently been suggested at the time of ablative surgery, for a higher success rate.^6^ The viability of immediate implant placement after tooth extraction performed at the time of ablative surgery or during routine dental evaluations before radiation therapy has also been demonstrated.^7^

The association of particulate bone grafts with dental implant placement during ablative surgery may be advantageous for regional volume maintenance.^7^ The use of particulate bone grafts avoids substantial loss of bone volume in areas with compromised bone anatomy, such as extraction sockets.^8^ Preliminary data showed that radiation delivered to bone defects during the repair process was enough to produce effects on bone microstructure, collagen maturation, and osteocytes.^9^ Ionizing radiation leads to an arrest of the bone repair process, characterized by morphological changes in the trabecular arrangement of the newly formed bone, and alterations in the osteocyte lacunar network and collagen maturation. Considering this, particulate bone grafts can benefit the bone repair process affected by radiation therapy. However, there is no evidence supporting the use of xenogeneic bone substitutes in areas that will be included in the radiation field for head and neck cancer treatment. Proper clinical management and reconstruction techniques with dental implants can greatly improve these patients rehabilitative potential.^10^ Therefore, this study aimed to assess the effect of a single dose of radiation therapy (15 Gy) on grafted and non-grafted defects on bone microarchitecture and collagen maturity.

## Methodology

### Study design

This study adhered to the normative guidelines of the National Council for Animal Control and Experimentation (CONCEA), a constituent of the Ministry of Science, Technology, and Innovation (MCTI; Law 11.794, 08/19/2008) in Brazil. The Bioethics Committee for Animal Experimentation of Universidade Federal de Uberlândia approved the experimental protocol (CEUA #093/17). The experimental study design followed all the Animal Research: Reporting of In Vivo Experiments (ARRIVE) guidelines. Sixteen male *Rattus norvegicus* (average weight of 300 g) were kept in cages with a 12-hour light-dark cycle and controlled temperature conditions (average 22±2° C). The diet consisted of standard laboratory pellets (Labina, Purina, Paulínia, SP, Brazil), and water was provided *ad libitum*.

Initially, the animals’ legs were trimmed and cleaned with a 0.2% chlorhexidine solution (Rioquimica, São José do Rio Preto, SP, Brazil) to prevent possible infections. Then, the animals were subjected to intraperitoneal anesthesia combining 100 mg/kg of 10% ketamine hydrochloride (Ketamina Agener^®^; Agener União Ltda, São Paulo, SP, Brazil) with 10 mg/kg of 2% xylazine hydrochloride (Rompum^®^ Bayer SA, São Paulo, SP, Brazil). Incisions of 3 cm were made, and bone defects were created in the femur using a spherical carbide burr (no. 8) (Angelus Prima Dental, Londrina, PR, Brazil) with 2.3 mm diameter. The perforation depth was limited to the rupture of the cortical bone. The right femur was left ungrafted, filled only by a blood clot (group “Clot”). The left femur was filled with inorganic particulate xenogeneic bone graft (group “Xenograft”) (Bio-Oss^®^ Small, Geistlich Pharma AG, Wolhusen, Switzerland). One gram of Bio-Oss^®^ (Small, Geistlich Pharma AG, Wolhusen, Switzerland) was equally divided in 20 portions of 0.05 g each for standardization. The graft quantity was carefully chosen to adequately fill the defect without compression or use of membranes. The periosteum was positioned over the surgical zone. Samples of the bone graft particles were placed in Eppendorf tubes in a sterile environment (laminar flow cabinet). After surgery, the animals were randomly divided into two groups (n = 8): (a) nRTX, without radiation therapy, and (b) RTX, with radiation therapy (single dose 15 Gy). Randomization was performed using the website *random.org*. All the procedures were performed by a single researcher (MSI).

### Radiation therapy

Seven days after the surgical procedure, the RTX group received a single -dose of 15 Gy ionizing radiation on both legs using a medical linear accelerator 6 MeV (Varian 600-C; Varian Medical System Inc., Palo Alto, California, USA). Animals were anesthetized by an intraperitoneal injection of 100 mg/kg ketamine and 70 mg/kg xylazine hydrochloride for immobilization and fixation with tape. A bolus measuring 1.5 cm in width was placed over the legs of all animals. This procedure lasted for 2 min. Animals in the nRTX group were anesthetized. All animals were euthanized 14 days after the bone grafting surgical procedure with an intravenous injection of 2.5% thiopental (150 mg/kg). The femurs were removed and immediately fixed in a 4% phosphate-buffered paraformaldehyde solution for 48h. The samples were first scanned using microcomputed tomography (micro-CT). Subsequently, the samples were decalcified in 4% ethylenediaminetetraacetic acid (EDTA) for 5 weeks, dehydrated with graded ethanol, and embedded in paraffin. Longitudinal histological sections of 5 μm were obtained and stained with hematoxylin and eosin (H&E) for qualitative analysis, Masson’s trichrome stain for histomorphometric analysis, and Picrosirius Red for quantitative collagen analysis.

### Micro-CT analysis

The samples were scanned using micro-CT^11,12^ (Skyscan 1176, Bruker, Kontich, Belgium) with a nominal isotropic voxel size of 9 μm (X-ray source 70 kV, 276 μA), using an aluminum filter (1 mm thickness) and 180º rotation with an angular increment of 0.4° and averaging of 2 frames. The reconstructions were performed using the nRecon software (version 1.6.10.1, SkyScan, Bruker, Belgium), following the parameters of smoothing (0), ring artifact reduction (12), and beam hardening (30%) for all samples. Each dataset was opened in DataViewer software (version 1.5 1.2. SkyScan; Bruker, Belgium) and the samples were rotated until the longitudinal axis of the femur was parallel to the horizontal plane. Coronal views were saved for further analysis. The images were analyzed using the CTAn software (version 1.14.4.1, SkyScan, Bruker, Belgium), using a standard threshold of 82-255 for the lot groups. The threshold was defined as the mean of the automatic threshold values (Otsu method) calculated from ten samples. For the Xenograft group analysis, the volume of the graft particles was estimated by applying a threshold value of 129–255. This value was obtained by calculating the predominant grey values in the histogram of the selected particles. The mean values obtained from 10 samples were used.

The bone volume fraction (BV/TV) results were obtained using a threshold of 82–128. The region of interest included only the area of newly formed bone using a circular predefined shape measuring 2.3 mm (lesion size), respecting the limits of the lesion top and bottom. To obtain a standard protocol for the analysis, the number of sections comprising the perforated cortical bone was assessed and 50 sections (approximately 0.45 mm) from the center were selected. The volume of interest (VOI) corresponded to the cortical portion of the lesion. A single operator performed all the analyses. The surgeons were blinded to the study groups. The bone volume fraction (BV/TV), trabecular thickness (Tb.Th), trabecular number (Tb.N), and trabecular separation (Tb.Sp) were analyzed.

### Bone matrix quantification and qualitative analysis

Masson’s trichrome-stained and hematoxylin-eosin (HE) sections were used to obtain histological images of the lesion, captured using a light microscope (Olympus BX61, Olympus Corporation, Tokyo, Japan) connected to a digital camera (Olympus DP80, Olympus Corporation, Tokyo, Japan) with an objective lens of 20x.

Masson’s trichrome-stained sections were evaluated for histomorphometric analysis. Hematoxylin and eosin HE sections were used for general histological analysis. Dedicated software (ImageJ 1.51k, National Institute of Health, USA) was used for quantitative analysis. The step-by-step methodology for image processing and analysis using the software has been detailed in previously published articles.^11,12^ A rectangular area of interest (AOI) drawn between the cortices and borders of the lesion was defined for each section. The software randomly generated a “grid,” providing an area of 3.675 pixels on top of each image. The grid was represented by intersecting lines with a distance of 40 μm between neighboring counting points. This grid configuration was determined to avoid over- and under-sampling of the histologic sections, as reported by Hartlev, et al.^13^ (2020). Each point was classified as (a) bone, (b) connective tissue, or, in the grafted groups, (c) bone substitute material. The graft particles were recognizable by their color and pattern. For the same AOI, the percentage of direct contact between the bone-substitute particles and bone was assessed in the xenograft groups. The values were expressed as a percentage of the bone-graft contact over the total surface of the particles. Intraclass correlation coefficients were estimated for intra-examiner agreement in 10 samples for quantitative histological measurements (ICC > 0.9) obtained before analysis.

### Collagen analysis

Collagen packing density was evaluated using Picrosirius Red-stained sections from five equal areas within the AOI of the lesion. Images were captured using a polarized light microscope (Nikon Eclipse Ti-S) with a 20x objective lens against a black background. Collagen analysis of each area was performed using the ImageJ software (ImageJ 1.51k, National Institute of Health, USA). The birefringence color changed from green to red (shorter to longer wavelengths) as the density of the collagen packing increased. Color thresholds were determined by individual pixel selection using the *Color Threshold* tool. Each pixel was categorized as follows: red, representing densely packed collagen; yellow, representing intermediately packed collagen; and green, representing loosely packed collagen. The results were obtained by calculating the number of pixels stained between an intensity threshold in bins determined by 8-bit hue values (0–25 for red, 26–52 for yellow, and 53–110 for green); modified from Smith and Barton^14^ (2014). The proportion of colors within birefringent tissue was normalized and compared between groups.

### Statistical analysis

Data were analyzed using the Shapiro-Wilk normality test. The influence of RTX and defect-filling treatments on bone morphometric parameters was assessed using a two-way analysis of variance (ANOVA). Tukey’s *post hoc* test was used for multiple comparisons when significant differences were detected. The percentage of bone volume grafts among the xenograft groups was compared using the unpaired Student’s t-test. In addition, an unpaired t-test was used to calculate the effect of RTX on bone-graft contact in histological sections. The differences were considered statistically significant at α=0.05. Statistical analyses were performed using Sigma Plot (v. 12, Systat Software, Inc., San Jose, California, USA).

## Results

The animals showed no signs of infection at the surgical site. A reduction in body weight (approximately 5%) was noted five days post-surgery in all animals. Radiation was well-tolerated and did not induce alopecia or weight loss until the day of euthanasia.

### Micro-CT analysis

**Figure 1:**
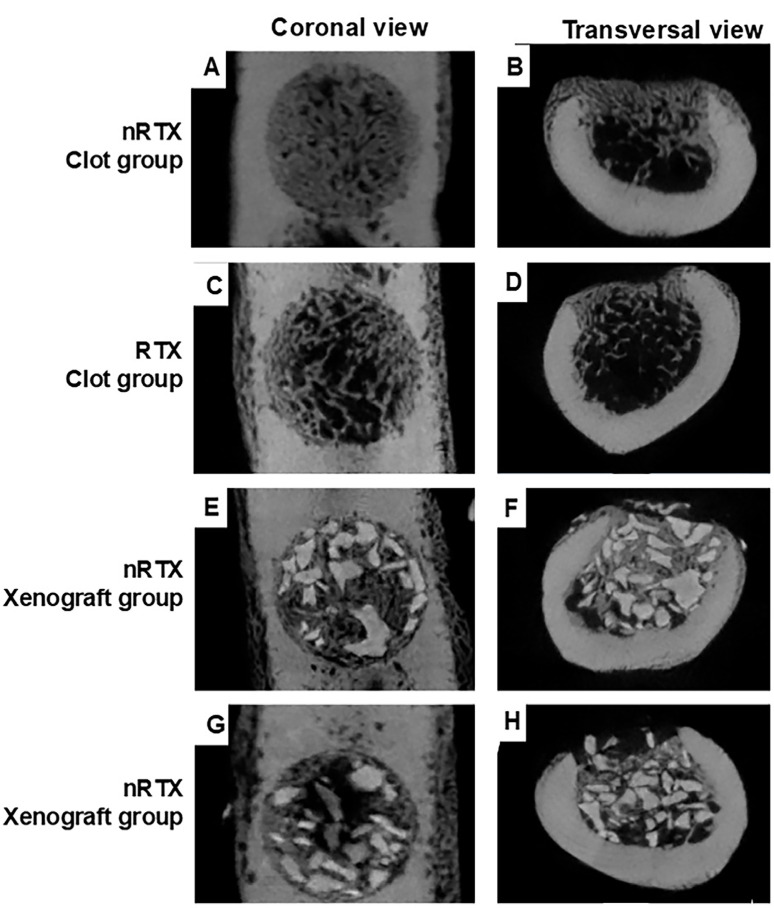
MicroCT images of the defect in Clot in nRTX (A: coronal view; B: transversal view) and RTX (C: coronal view; D: transversal view) groups and Xenograft in nRTX (E: coronal view; F: transversal view) and RTX (G: coronal view; H: transversal view) groups.

Figure 1 illustrates the raw images of microCT analysis. The means and standard deviations of the micro-CT parameters are listed in Table 1. Radiation therapy resulted in lower BV/TV (P=0.005) and Tb. N (P<0.001) values in both the Clot and Xenograft groups, whereas a decrease in Tb.Sp (P<0.001) was observed only in the Clot group. Defects filled with xenografts showed a larger Tb.N (P<0.001) in both nRTX and RTX groups. In contrast, the clot group demonstrated increased BV/TV (P<0.001) and Tb.Th (P<0.001), regardless of radiation therapy treatment, whereas Tb.Sp was larger (P<0.001) only in the RTX group. There was no statistically significant difference (P=0.114) in the percentage of graft particle volume between the groups.

**Table 1:** Mean and standard deviation values of the micro-CT analysis

	nRTX	RTX
**Bone volume fraction (BV/TV)**		
Clot	59.5 (5.4)^Aa^	51.1 (10.1)^Ab^
Xenograft	45.0 (7.9)^Ba^	41.1 (4.5)^Ba^
**Trabecular thickness (Tb.Th)**		
Clot	0.09 (0.01)^Aa^	0.11 (0.02)^Aa^
Xenograft	0.06 (0.01)^Ba^	0.05 (0.00)^Ba^
**Trabecular number (Tb.N)**		
Clot	6.2 (0.5)^Aa^	4.6 (1.1)^Ab^
Xenograft	7.9 (0.4)^Ba^	7.4 (0.6)^Bb^
**Trabecular separation (Tb.Sp)**		
Clot	0.09 (0.01)^Aa^	0.19 (0.07)^Ab^
Xenograft	0.10 (0.01)^Aa^	0.11 (0.01)^Ba^

Different uppercase letters indicate significant difference between the rows; different lowercase letters indicate significant difference the columns.

### Histomorphometric analysis and histological findings

**Figure 2:**
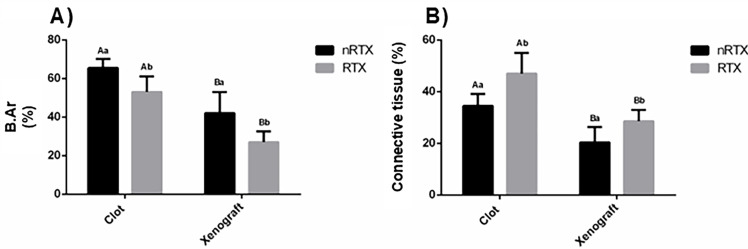
Histograms showing the histomorphometric results (mean±SD). Percentage of new bone area (A) and soft tissue (B) among the groups.

Figure 2 presents the results of histomorphometric analysis. The amount of woven bone formed significantly reduced in the RTX (P<0.001) and xenograft groups (P<0.001). The presence of connective tissue was also higher in these groups (P<0.05). No interaction was observed between the two factors (radiation therapy and defect filling) (P=0.7).

**Figure 3:**
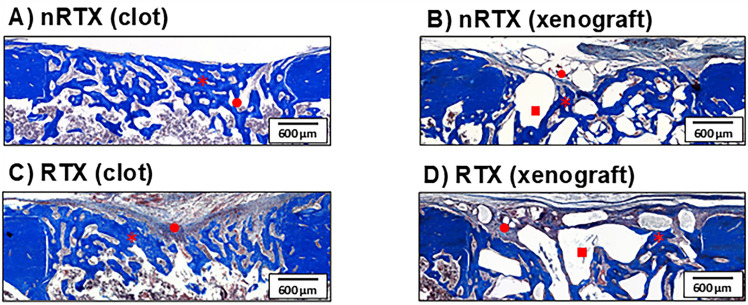
Photomicrographs of the cortical portion of the defect in Clot (A: nRTX; C: RTX) and Xenograft (B: nRTX; D: RTX) groups. (Masson’s Trichrome stained; asterisc: bone; square: grafted material; circle: granulation tissue).

Histological observations confirmed the presence of defects in all groups. Qualitative histological analysis of decalcified sections from the control group (nRTX and non-grafted samples) demonstrated the presence of bridging bone formation across the defect (Figure 3). In the non-grafted irradiated samples, it was verified that the trabeculae of newly formed bone lacked bridge formation (Figures 3A and 3C). In the nRTX (xenograft) group, the newly formed tissue reached the center of the defect, extending from the periphery of the defect and along the direct surface of the grafting material granules (Figure 3B). In the RTX (xenograft) group, thinner trabeculae of the newly formed bone extended along the defect, presenting low interconnectivity and circulating grafting material granules (Figure 3D).

**Figure 4:**
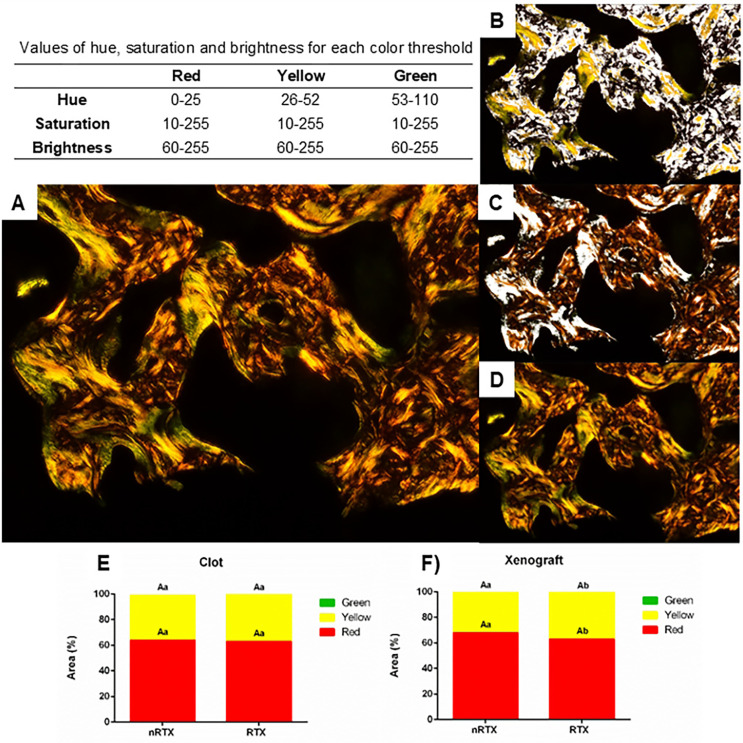
Quantification analysis of fibrillar collagen levels (mean±SD). (A) Polarized photomicrograph of the woven bone; (B) Red pixels selected (white area) by color threshold; (C) Yellow pixels selected (white area); (D) Green pixels selected (white area); (E) The normalized proportion of green, yellow, and red birefringent collagen bundles in Clot groups; (F) The normalized proportion of green, yellow, and red birefringent collagen bundles in Xenograft groups. (PicroSirius Red stained) Different uppercase letters indicate significant difference between Clot and Xenograft groups; different lowercase letters indicate significant difference between nRTX and RTX groups

The extent of collagen packing was analyzed as a proportion of the collagen area (Figure 4). Intermediately (yellow) and densely packed collagen (red) were predominant among the groups. A minute fraction of the collagen area (<1%) was occupied by loosely packed collagen (green) in all groups. The proportions of densely packed and intermediately packed collagen were similar (P>0.05) in all groups filled with clots. However, radiation therapy decreased the amount of densely packed collagen in the xenograft group (P<0.05).

**Figure 5:**
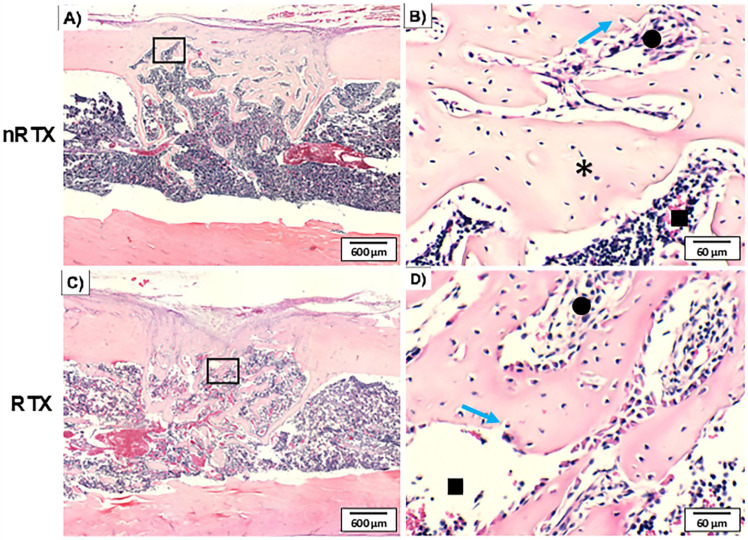
Photomicrograph of the non-grafted groups 14 days after surgery. The healing stages of the lesions were evaluated from the longitudinal femur sections. An approximated view shows the woven bone formed in the cortical portion of the lesion. Thinner trabeculae with granulation tissue between them were observed in the RTX groups. An impairment of the defect closure was also noted. (H&E stained; asterisk: bone; square: medullary tissue; circle: granulation tissue; blue arrow: osteocyte being embedded).

In the Clot group (Figure 5), nRTX histological sections showed predominantly trabecular bone, and defect closure was almost complete. The periosteum was well-organized above the newly formed bone. The cortical portion of the lesion in the irradiated group (RTX) was filled with woven bone containing more elongated trabecular bone. Granulation and medullary tissue between the trabeculae were common. The healing pattern showed disorganized bone formation bridging the lesion cortical borders. Blood vessels were observed more frequently in the nRTX group.

**Figure 6:**
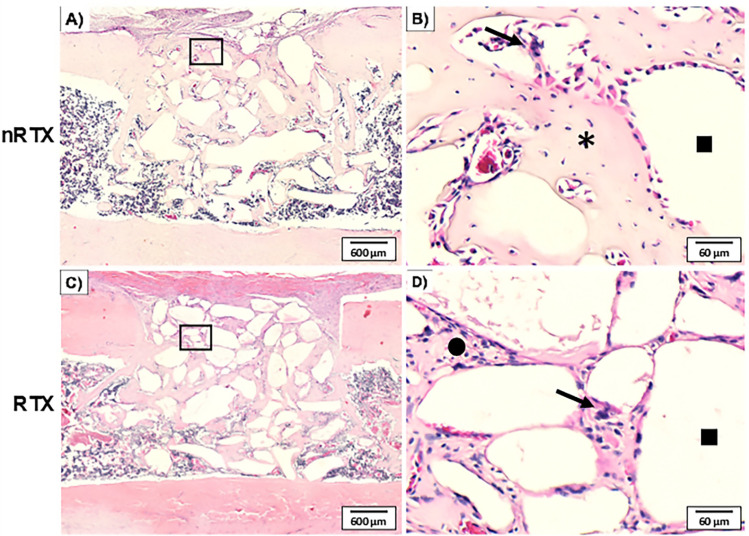
Photomicrograph of the grafted groups 14 days after the surgery. Overview of the longitudinal femur sections of the nRTX (A) and RTX (C). An approximated view shows the remodeling process around the particles. Note the bone formation in the nRTX groups (B) and the presence of granulation tissue in the RTX groups (D). (H&E stained; asterisc: bone; square: grafted material; circle: granulation tissue). Multinucleated cells (black arrow) were observed on the biomaterial surface.

Figure 6 shows the grafted sample sections. Histological sections revealed a larger periosteal invagination in the AOI of the grafted groups. In the nRTX group, the cortical portion of the lesion showed mostly primary bone (woven bone) surrounding the particles, and a more mature healing pattern aimed at wound closure. In contrast, connective tissue was more evident in the RTX group, mainly in the external portion of the AOI. However, primary bone involvement of the particles was evident in the AOI near the medullary area of the lesion and within the cavity. Multinucleated cells were observed more frequently in the grafted groups.

## Discussion

This study showed that a single dose of irradiation (15 Gy) impaired bone healing, which was characterized by alterations in the woven bone microstructure. This effect was observed for both grafted and non-grafted defects. The bone repair process in irradiated areas has been extensively evaluated. However, few studies have focused on the effect of radiation therapy on the ongoing healing of surgical sites.^9,15,16^ This is assumed to have great relevance considering the possibility of defect reconstruction and dental implant osseointegration before initiating radiation therapy sessions.

The literature shows a tendency towards implant placement during the same intervention as ablative surgery to avoid surgical procedures in irradiated tissues and to shorten the prosthetic rehabilitation time.^10^ This approach usually involves free-flap jaw reconstruction and has shown to be a feasible option for prosthetic rehabilitation in oral cancer.^17,18^ A retrospective study involving 210 patients revealed a higher success rate of implant osseointegration in irradiated patients when implants were placed immediately during flap transfer than when a delayed placement was adopted (86 versus 64%).^19^ Evidence of increased damage when radiation therapy was delivered preoperatively and minimal effects on bone healing when applied postoperatively, supports this clinical finding.^16,20^Planning the rehabilitation treatment before radiation therapy sessions provides more time for the integration of the graft and the implant.^21^ Therefore, the investigation of radiation therapy effect on bone repair in defects filled with particulate bone grafts has been based on these findings.

In a previous study by our group,^9^ 30 Gy delivered 2 weeks postoperatively did not affect the bone volume fraction of defects in rabbits. Interestingly, a decreased bone volume fraction was evident in this experiment after 7 days of irradiation. To explain these findings, the mechanisms involved in the repair process and the timing of radiation delivery must be considered. Bone healing of burr “hole” defects in long bones consists of two healing stages. The first is a phase of predominant woven bone formation along the lateral periosteal bridging the cortical bone, followed by a second phase of woven bone replacement with lamellar bone. In rats, the first phase was observed until day 7. Subsequently, the woven bone is remodeled into lamellar bone to achieve defect closure, and resorption of the woven bone inside the medullary cavity is observed.^22^ Associating the healing phase with the irradiation timing is crucial for a proper result interpretation. It has been demonstrated that the effect of radiation therapy on bone healing varies with the degree of tissue maturation. The interval of four days or more between the surgery and the postoperative irradiation dramatically decreased the deleterious effect of radiation therapy on bone healing in rats. At the same time, when radiation therapy had been delivered before the surgical intervention or in the early postoperative period (within three days), a decrease in bone formation was observed.^16^ Thus, divergent results among studies might be due to the distinct intervals between the surgery and the postoperative irradiation, as well as the use of different animal models.

The effect observed on the bone microstructure could probably be observed within a short period after irradiation, because radiation therapy mainly affects the proliferative capacity of osteogenic precursor cells.^23^ The lesion poses a challenge for the physiological environment, which requires the recruitment and differentiation of mesenchymal cells,^24^ affected by high doses of ionizing radiation during this process. This mechanism involves metabolism acceleration in both hard and connective tissues to enhance tissue healing and local tissue defensive reactions.^22^ Without this trigger, the radiation effect on the bone mineralized portion probably would take longer to appear as reported previously.^25^The ionizing radiation was delivered in a single dose of 15 Gy. It promotes the rapid death of most cells sensitive to ionizing radiation, whereas fractionated applications induce a dose- and delay-dependent response.^26^ The most commonly used protocol for head and neck cancer, when radiation therapy is administered alone, is 2 Gy in a single fraction per day, five days a week, for six to seven weeks. The dose used in this study corresponds to a total dose of approximately 64 Gy, when using multiple incremental doses of 2 Gy. Thus, it is compatible with the protocol conventionally adopted in radiation therapy of head and neck and with the minimum irradiation (60 Gy).^26,27^ Fractionated dose delivery in animal experiments was not suitable, due to the tight schedule of the linear accelerator device used for treatment and anesthetic difficulties related to animal experimentation.

Regarding grafted defects, radiation did not interrupt the bone integration with the grafted particles. In the histomorphometric analysis, only the actual bone area was measured, excluding the area of the graft particles. Deproteinized bovine bone mineral particles (DBBM) degrade slowly. These bone graft particles have been found 4 to 10 years after implantation.^28^ This characteristic allows for the volume maintenance of the grafted areas over time, which is advantageous, especially in areas of affect function and esthetics by bone dehiscences.^8^ Thus, it was expected to find a lower percentage of newly formed bone in grafted groups during this initial healing period. Bone substitute material whose complete resorption takes long periods results in a reduction of newly formed bone, since the particle occupies continued space.^29^ An ideal experimental model to assess bone formation in grafted areas for longer periods involves creating critical defects without spontaneous bone healing during the animal’s lifetime. Larger defects in rat femur may lead to fractures; thus, critical calvarial defects are the most frequently used. However, radiation delivery of 15 Gy in a single dose was not possible in this area, making this model unfeasible for our study. Our defects were analyzed after a 14-day period, during which spontaneous healing of the femur defects did not occur. Consequently, there was no need to employ a larger defect size to evaluate the effects of radiation therapy on defect healing.

To enable a direct comparison between the grafted and non-grafted groups without the interference of the volume occupied by the particles, micro-CT analysis of the bone volume fraction was performed by subtracting the graft volume from the total volume. Results revealed an increased percentage of bone volume in both the RTX and nRTX groups. The morphometric results from the micro-CT agreed with the histomorphometric findings. Bio-Oss^®^ particles (Geistlich Pharma AG, Wolhusen, Switzerland) were visually distinguished from newly formed bone due to their different gray levels. The threshold for particle segmentation was determined by selecting the graft alone and performing further histogram analysis on ten samples to minimize any possible bias that may arise from the micro-CT methodological analysis. Although misleading measurements at the bone-graft interface might occur in the micro-CT analysis, histological analysis was also performed to ensure the results reliability. Histological analysis showed that the osteoconductive property of the xenogeneic granules was maintained with no signs of necrosis after irradiation. In addition, irradiation delivery did not affect bone-graft incorporation, as demonstrated by the percentage of particle surface in contact with the bone after radiation therapy.

Only a few studies have evaluated the influence of postoperative irradiation on bone properties around particulate bone substitutes.^26,30-32^ Malard, et al.^33^ (2005) investigated the possibility of bone reconstruction using macroporous biphasic calcium phosphate (CaP) inserted 3 weeks before radiation therapy and associated with autologous bone marrow (BM) grafts injected after irradiation. The results showed that CaP osseointegrated even without the marrow graft association. Histological analysis revealed the absence of fibrous tissue between the bone and the implanted CaP, similar to this study findings. The authors suggested that this integration was initiated before irradiation and was preserved, contributing to bone bonding and stability. The same behavior has been noted in the osseointegration of implants coated with hydroxyapatite: the more organized the peri-implant bone at irradiation onset, the less the radiation damage on bone formation.^21^ However, since the bone remodeling of irradiated bone is altered in a long-term period,^34^ future studies should evaluate the behavior of the grafted tissues over time, as well as the implant osseointegration in these areas. Nonetheless, this study findings are promising, mainly because osteoconductive biomaterials can enhance bone deposition in unfavorable regions,^35^ which has been shown to be preserved after irradiation.

There is extensive literature regarding the osteoconductive property of xenogeneic bone grafts. From a clinical perspective, its association with vascularized free flaps might be an option for larger reconstructions, considering that the particles serve as a scaffold for osteogenic cells,^36^ supporting the consolidation of the free flap and host before radiation therapy. Oral rehabilitation supported by implants can be improved in specific cases, when bone substitutes aid in minimizing bone volume loss over time, as demonstrated in non-irradiated bones. A lower survival rate of implants installed in irradiated grafted bone than in native bone has been reported.^37^ These results were expected because of the increased complexity of the anatomical sites in cases requiring grafting procedures. However, a recent systematic review demonstrated that the survival rate for dental implants placed in both native and grafted bone during ablative surgery remained high (90.4%).^38^

Another fundamental aspect that requires further investigation to ensure the predictability of oncological treatments is the backscattering effect in the presence of bone substitute materials. The particulate graft used in this experiment was a deproteinized bovine bone material, with crystalline hydroxyapatite as a constituent. Hydroxyapatite showed a minute effect, whereas poly-L-lactide PLA (bioabsorbable polymer) led to minimal radiation dose inhomogeneity for both photons and electrons.^39^ The biological implications of the backscatter and attenuation effects caused by the presence of bone substitute materials should be further evaluated.

Collagen crosslinking is considered a key predictor of bone fracture risks.^40^Moreover, the early post-radiotherapeutic increase in osteoclastic activity is followed by the long-term depletion of local osteoclasts. Consequently, collagen hypermineralization and hyperorientation occur.^34^ This effect has been reported in mature bones. In newly formed bone, a decrease in collagen density has been demonstrated after a single dose of 30 Gy.^9^ In this study, 15 Gy delivered post-surgically did not affect collagen-packing density in defects filled with clots. However, a small percentage of densely packed collagen was observed in irradiated grafted defects. This finding can be attributed to the lower proportion of soft and hard tissues in the grafted defects than that in the clotted group. Quantification of fibrillar collagen levels after Picrosirius Red staining involved both mineralized and non-mineralized tissues. Thus, because the components of the irradiated wound have different degrees of sensitivity,^41^ it is impossible to assume that the collagen packing density, specifically that of the newly formed bone, was affected. Histological sections revealed greater periosteal collapse inside the grafted groups AOI. Ionizing radiation may have a major initial effect on periosteal collagen organization during the healing phase. However, further research is required to validate this hypothesis.

This study presents drawbacks that must be considered in data interpretation. The evaluation period chosen represents a short-term healing process (14 days), and longer periods may provide more detailed information regarding the healing evolution in grafted and non-grafted areas. Non-critical defects in rats’ long bones require 6-8 weeks to achieve bone tissue complete repair.^42,43^ So, additional studies are necessary to evaluate the radiation therapy effect after grafting procedure with osteoconductive bone substitutes in the long term. However, this study provides valuable information regarding the radiation therapy effect in the short term, which is the most critical moment for evaluating the repair process, as earlier healing phases present greater tendencies to detect the effects of factors external to the healing process.^42,43^Furthermore, the evaluation of the radiation therapy effect in more structural bone proteins expression and on the regulation of signaling pathway related with bone metabolism during the healing phases is necessary. Besides, the oral environment was not reproduced in this study, but promising results related to the osteconductive property of the bone graft particles applied before irradiation should encourage more efforts to investigate its application in clinical conditions before the radiation therapy begins.

## Conclusion

In conclusion, the preoperative delivery of 15 Gy led to a delay in bone repair, as demonstrated by the smaller amount of newly formed bone in both grafted and non-grafted defects. However, graft particle integration with the newly formed bone was maintained even after 7 days of irradiation.
